# Biogenesis of TNF‐α‐insights into proteostasis and inflammation

**DOI:** 10.1111/febs.70553

**Published:** 2026-04-25

**Authors:** Bailasan Haidar, Céline Philippe, Eric Chevet, Jiří Zahradník

**Affiliations:** ^1^ INSERM U1242, University of Rennes France; ^2^ First Faculty of Medicine, BIOCEV, Charles University Vestec‐Prague Czech Republic; ^3^ Centre de lutte contre le Cancer Eugène Marquis Rennes France

**Keywords:** autophagy, cytokine signalling, endoplasmic reticulum stress, ER‐associated degradation, inflammation, NF‐κB pathway, protein aggregation, proteostasis network, tumor necrosis factor‐alpha, unfolded protein response

## Abstract

Tumour necrosis factor‐α (TNF‐α) is a central pro‐inflammatory cytokine whose biogenesis, secretion, and signalling are tightly interconnected with cellular protein‐quality control systems. Current evidence shows that TNF‐α maturation, co‐translational and post‐modifications, ER‐luminal folding and trimerisation, Golgi trafficking, and ectodomain shedding by ADAM17 are constrained by ER chaperones and ER‐associated degradation (ERAD). Furthermore, TNF‐α signalling reciprocally interfaces with the proteostasis network (PN) largely through inflammatory stress pathways such as NF‐κB‐dependent transcriptional control of chaperones, ubiquitin‐proteasome components, and autophagy regulators. However, dysregulation of this bidirectional crosstalk mechanistically contributes to disease, including chronic inflammatory disorders, cancer, and degenerative diseases. In this study we provide a synthesis of the current literature on pathways related to protein homeostasis control that determines whether TNF‐α exposure is adaptive or proteotoxic. We also discuss the translational implications this could have by including rational combinations of TNF‐α targeted blockers with PN modulators (chemical chaperones, proteasome or autophagy modulators), which reduce the proteotoxic burden. Therefore, understanding the crosstalk between TNF‐α signalling and components of the PN system promises new mechanistic insights and translational targets for TNF‐α‐driven diseases.

## Graphical abstract

TNF‐α biogenesis, trafficking, and signalling are tightly and reciprocally coupled to cellular proteostasis systems, including ER chaperones and endoplasmic reticulum‐associated degradation. This bidirectional crosstalk determines whether TNF‐α responses are adaptive or proteotoxic. Its dysregulation contributes to inflammatory, cancerous, and degenerative diseases.

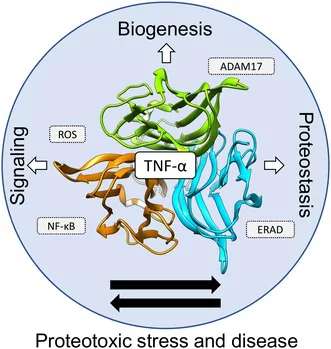

**Abbreviations**
ER: endoplasmic reticulum
ERAD: ER‐associated degradation
NF‐κB: nuclear factor kappa B
PDI: protein disulphide isomerase
PN: proteostasis network
ROS: reactive oxygen species
SG: stress granules
TNF‐α: tumor necrosis factor‐α
UPR: unfolded protein response

## Introduction

Tumour necrosis factor alpha (TNF‐α) is a cytokine that plays a central role in inflammation and immune regulation, and as such, has been thoroughly investigated. Its biosynthesis is tightly controlled, and relies on the coordinated action of the cellular protein quality control machinery, including proper folding, glycosylation, and trafficking through the secretory pathway. Notably, TNF‐α is not only a key player in immune regulation but also actively shapes it through its downstream‐signalling pathways. TNF‐α can modulate the expression and activity of various components of the protein homeostasis machinery, also known as proteostasis, thereby influencing the cell's capacity to manage misfolded proteins and maintain proteostasis under stressful or pathological conditions. In this review, we examine the bidirectional relationship between TNF‐α and the proteostasis network (PN), thus distinguishing the direct interactions between TNF‐α and PN components, from indirect effects that are mediated by inflammatory stress pathways, including ROS, NF‐κB signalling, and ER stress. We will also discuss physiological and pathological events, such as chronic inflammation and neurodegeneration, where TNF‐α‐mediated proteostasis dysregulation plays a critical role. The initial discovery of tumour necrosis factor identified this protein as an endotoxin‐induced agent causing tumour necrosis in mice [1]. TNF‐α is a pleiotropic cytokine, particularly notable among its TNF superfamily for its widespread expression and potent biological effects in both physiological and pathological contexts, including autoimmune diseases and cancers. It has emerged as a central mediator of inflammation, immune regulation, and cellular stress responses, acting primarily through two receptors: TNFR1 (also known as TNFRSF1A, CD120a, or p55) and TNFR2 (also known as TNFRSF1B, CD120b, or p75) [2]. Overall, the TNF superfamily comprises over 19 ligands and 29 receptors that regulate diverse cellular functions, such as proliferation, apoptosis, differentiation and inflammation. While they share structural homology, different members exhibit tissue‐specific expression and distinct downstream signalling profiles. Members such as Fas ligand (FasL), TRAIL, and CD40L have been implicated not only in immune cell communication but also in regulating stress responses, autophagy, and cell death. This highlights their role as modulators of the cellular proteostasis network [3].

Eukaryotic protein homeostasis, also known as proteostasis, refers to the dynamic regulation of the cellular proteome to maintain proper protein folding, function, and turnover [4]. Central to this process is the PN, a coordinated system of molecular chaperones, folding enzymes, trafficking components, and degradation pathways that controls and protects protein integrity throughout the cell. Computational analysis of the PN's architectural backbone reflected its deep conservation across evolution of the protein homeostasis network (from bacteria to mammals), which represents a fundamental requirement for cellular viability, regardless of ecological niche or complexity of the organism [5].

The endoplasmic reticulum (ER) plays a critical role in cellular proteostasis, as about one‐third of the total proteome passes through this compartment (mostly secretory and transmembrane proteins). In the ER, newly synthesised polypeptides undergo folding and post‐translational modifications operated by ER‐resident chaperones and folding enzymes. To ensure protein quality, the ER utilises three major surveillance mechanisms: [1] ER quality control, which retains misfolded proteins to promote refolding; [2] ER‐associated degradation (ERAD), which retro‐translocates terminally misfolded proteins for ubiquitin‐proteasome degradation; and [3] the unfolded protein response (UPR), a signalling network initiated by three ER‐resident transmembrane sensors (IRE1α, PERK and ATF6α). IRE1α (an ER‐localised kinase/endoribonuclease [IRE1]) activates XBP1s and can engage stress kinases (e.g. JNK1), PERK phosphorylates eIF2α to transiently attenuate translation, and ATF6α (ATF6) is trafficked to the Golgi for proteolytic activation into a transcription factor that upregulates chaperones and ERAD components [6]. Together, these pathways prevent proteotoxic stress by either repairing or disposing of folding defects [7]. If proteostasis is profoundly altered, persistent ER stress and UPR activation can drive inflammation and disease. Prolonged UPR signalling induces selective autophagy of ER (ER‐phagy) to clear aggregated proteins and damaged ER, further linking ER proteostasis to cellular stress responses [8, 9]. Through these pathways, the ER actively shapes the proteome and prevents the buildup of dysfunctional proteins [7].

Failure of these adaptive mechanisms to achieve their goals contributes to numerous pathological conditions, including age‐related, degenerative, neoplastic, metabolic, and immunological diseases [10, 11, 12]. A balanced PN dynamically counteracts protein misfolding and subsequent cellular damage in response to stressors like heat, oxidative stress, inflammation, or infections by pathogens, allowing cells to cope with challenging conditions. This also happens in a tissue‐specific manner, including differential activation of PN components. Different cell types carry their different PNs to their specific functional and environmental demands, leading to ‘differential PN’ across tissues, whether secretory vs. non‐secretory tissues (e.g. glands vs. connective tissues), metabolic demands (e.g. muscle cells vs. adipocytes), and stress exposure (e.g. skin vs. internal organs). For example, terminal differentiation of B cells into antibody‐secreting plasma cells and exocrine pancreatic cells requires XBP1s‐driven expansion of ER and secretory machinery to sustain massive immunoglobin secretion [13]. Muscle tissues rely on specialised chaperone/co‐chaperone systems (e.g. BAG3–Hsp70) to maintain proteostasis of the muscle sarcomeres, and loss‐of‐function BAG3 mutations cause protein‐aggregation myopathies [14]. Similarly, mutations in rhodopsin like P23H lead to ER retention and misfolding in the photoreceptors, activating the UPR and degradation pathways and causing retinal degeneration in retinitis pigmentosa [15]. Together, these examples show how tissues deploy distinct PN repertoires to meet their functional and environmental demands, with failures in node‐specific components producing tissue‐selective disease phenotypes. Importantly, cells under proteotoxic stress can transmit signals to their surroundings and/or distant tissues through endocrine and paracrine factors, including cytokines, hormones, and any other secreted peptides. These signals engage systemic immune defense responses and provide a broader level of intercellular coordination. However, whether such immune‐mediated communication can support systemic proteostasis remains an open and compelling question for future research. Emerging work shows that proteostasis can be regulated cell non‐autonomously [16]. For example, neuronally induced XBP1s activate ER‐UPR programmes and stress resistance in distal tissues, and systemic stress‐signalling pathways coordinate proteostasis between organs, by which immune signals might relay a proteostatic state organism‐wide [17, 18]. More generally, inflammation itself can induce ER stress and engage the unfolded protein response: pro‐inflammatory cytokines such as TNF‐α, IL‐1β, and IL‐6 have been shown to activate IRE1, PERK and ATF6 branches of the UPR, creating bidirectional crosstalk between inflammatory signalling and proteostasis [19]. Consistent with cytokine‐driven UPR crosstalk, Yap et al. showed that TNF‐α selectively activates the IRE1α–XBP1 pathway in primary human airway smooth muscle cells in a dose‐ and time‐dependent manner, an effect linked to TNF‐dependent ROS production [20].

Intriguingly, TNF‐α signalling both depends on and actively modulates quality‐control systems, creating a bidirectional crosstalk between inflammatory signalling and proteome homeostasis. Recent studies have shown that TNF‐α signalling modulates the activity of several molecular machineries associated with PN maintenance, such as autophagic flux and ubiquitin/proteasome protein degradation pathways [21, 22]. These findings suggest that TNF‐α impacts proteostasis largely through crosstalk between inflammatory signalling and protein‐degradation machinery rather than through direct regulatory control.

## TNF‐α biogenesis and the proteostasis network

### Transcriptional and post‐transcriptional regulation of TNF‐α expression

The human TNF‐α gene resides in the class III region of the major histocompatibility complex on chromosome 6 [23]. Notably, chromatin remodeling of the TNF‐α gene does not occur before stimulation, yet its promoter region has the prerequisite developmentally established histone acetylation required for its rapid expression [24, 25].

Stimuli, such as LPS (lipopolysaccharide) or IL‐1β, trigger rapid TNF‐α expression within minutes [25]. Histone acetyltransferases (CBP/p300) and the mediator complex remodel chromatin, facilitating Pol II accessibility at the promoter region and subsequent transcription initiation and elongation [25]. In both macrophages and lymphocytes, stimulus‐specific transcription factor complexes assemble on overlapping a TNF‐α promoter motif. These complexes include NFAT, ATF‐2/c‐Jun, Sp1, Ets family members, and cAMP‐response element‐binding factors [26]. In T cells, calcium‐dependent activation of calcineurin leads to dephosphorylation and nuclear translocation of NFAT proteins (especially NFATp). Dephosphorylated NFAT rapidly translocates to the nucleus and drives TNF transcription within minutes of stimulation [27]. In contrast, upon viral infection or LPS stimulus, nuclear NFAT is low, and Sp1 and Ets/Elk proteins bind their respective sites at the TNF‐α promoter to promote its expression [27, 28]. Although the distal promoter harbours several NF‐κB (nuclear factor kappa B)‐like motifs (κB1–κB4), classical NF‐κB binding is neither necessary nor sufficient for primary TNF‐α induction in macrophages or T cells. NF‐κB may instead contribute to post‐induction events (e.g. chromatin remodeling, LPS tolerance), while initial promoter activation relies predominantly on NFAT/ATF‐2/c‐Jun complexes [27]. By contrast, in LPS‐stimulated macrophages, the selective nuclear accumulation of NF‐κB p50 homodimers preferentially binds three κB elements in the murine TNF‐α promoter (κB1, κB2a, κB3). The binding of p50 homodimers appears to attenuate TNF‐α gene transcription and thereby limit the pro‐inflammatory response of activated macrophages [29].

Co‐transcriptionally, the primary TNF‐α transcript is spliced into a ~ 1.7 kb mRNA comprising four exons and its 3′‐untranslated region (3′‐UTR) contains AU‐rich elements (AREs) that render the mRNA unstable. Tristetraprolin (TTP, ZFP36) destabilises TNF‐α mRNA through its binding to AU‐rich elements; this post‐transcriptional feedback downregulates TNF‐α and is essential for resolving inflammation [30, 31]. Although alternative splicing of TNF‐α mRNA is rare, regulatory genetic variants such as rs1800629 in the TNF‐α promoter significantly influence expression levels. Such a mutation increases stimulus‐induced TNF transcription and is associated with inflammatory and autoimmune conditions (e.g. asthma, coeliac disease, metabolic syndrome) [32, 33].

Translation of TNF‐α begins on ER‐bound ribosomes. It uses the Sec61 translocon to be translocated into the ER and an internal signal‐anchor sequence (approximate residues 17–37) mediates co‐translational insertion into the ER membrane [34, 35]. This step targets TNF‐α for folding and maturation and links protein synthesis to ER quality control.

### Protein Folding & Quality Control

A bidirectional axis exists between inflammation and quality control. Essentially, inflammatory signals induce TNF‐α expression and secretion. TNF‐α is initially synthesised as a type II transmembrane precursor (pro‐TNF‐α), which must undergo folding, quality control, and trimerisation in the ER to become functionally active. Pro‐TNF‐α is then proteolytically cleaved in its juxtamembrane extracellular region to release a soluble ~ 17 kDa cytokine (sTNF‐α). Importantly, the balance between membrane‐bound and soluble TNF has important functional consequences because tmTNF mediates reverse signalling and TNFR2‐biased signalling, whereas sTNF preferentially activates TNFR1‐driven pro‐inflammatory pathways [36].

Upon synthesis on ER‐bound ribosomes, the nascent pro‐TNF‐α polypeptide immediately engages cytosolic chaperones: HSP40 (DNAJB1) recognises hydrophobic clusters in the N‐terminal tail (residues ~ 1–30) and recruits HSP70 (HSPA1A), whose ATP‐driven clamp prevents off‐pathway aggregation until the internal signal‐anchor (residues ~ 17–37) enters the Sec61 channel [37]. Unlike cleavable signal peptides, this signal–anchor both arrests translocation and anchors the N‐terminus in the cytosol, while the C‐terminal TNF‐α homology domain (~ 193 aa) enters the ER lumen, ensuring that it emerges into the ER lumen in a folding‐competent conformation [35]. Within the ER lumen, nascent TNF‐α interacts with general and lectin chaperones, such as BiP, an ER‐resident chaperone (Grp78), which binds exposed hydrophobic regions, preventing aggregation [38].

Once the C‐terminal TNF‐α homology domain enters the ER lumen, protein disulfide isomerase (PDI)‐A1 catalyses disulfide shuffling. The N‐terminal Cys69 of TNF‐α attack the PDI disulfide, forming a mixed PDI–TNF–α disulfide intermediate. Intramolecular attack by Cys101 then resolves this intermediate, creating the native Cys69‐Cys101 bond. Finally, PDI is re‐oxidised by ER oxidoreductin‐1α to complete the cycle. Importantly, it was shown that PDI‐A1 inhibition significantly reduces TNF‐α secretion and suppresses the TNF‐α‐induced inflammatory response of macrophages [39]. Largely, such control of misfolded or unassembled monomers is recognised by ERAD machinery, ensuring only correctly folded TNF‐α exits the ER [40]. ERAD is the ER quality‐control pathway that recognises persistently misfolded luminal and membrane proteins, directs them to membrane‐embedded ubiquitin ligases such as the SEL1L‐HRD1 complex, for polyubiquitination, and mediates their retro‐translocation and proteasomal degradation in the cytosol. This ordered process prevents aggregation and inappropriate surface delivery of defective polypeptides. Recent primary studies demonstrate that ERAD components actively regulate substrate selection, retro translocation efficiency, and the metabolic fitness of immune cells during inflammatory stress. These findings establish ERAD as a dynamic modulator of proteostasis rather than a passive disposal system [41, 42].

**Fig. 1:**
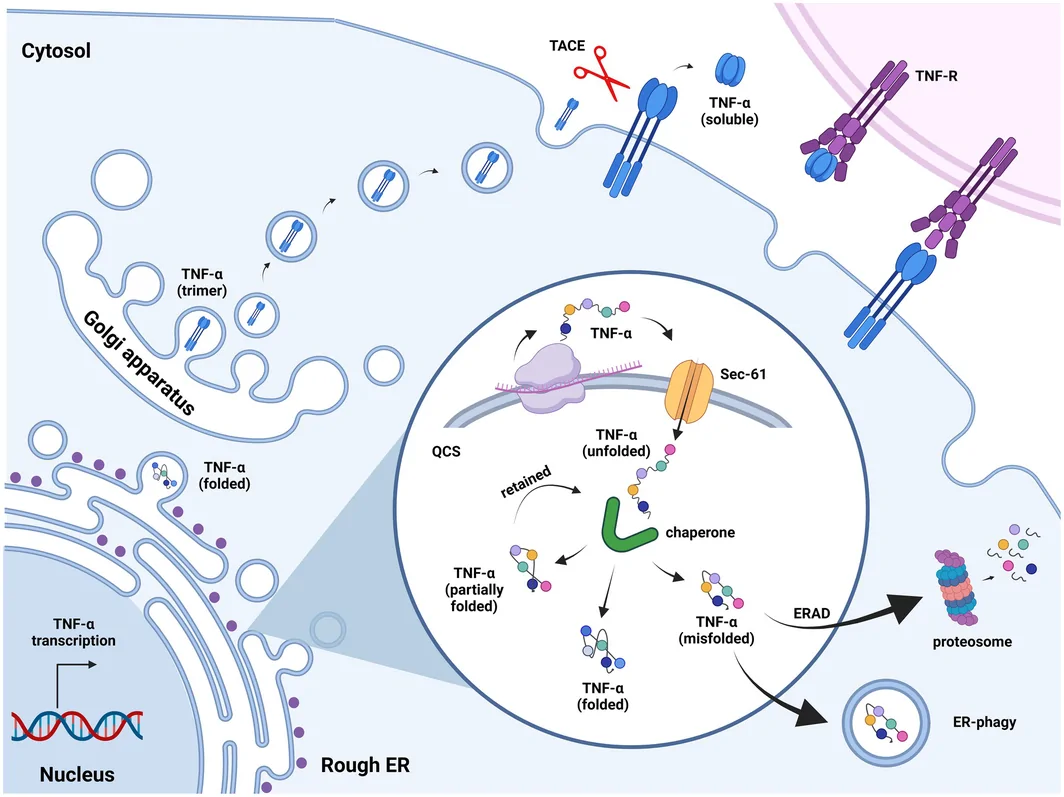
TNF‐α biogenesis, secretion and signalling. TNF‐α undergoes transcription in the nucleus, and is exported as mRNA to the cytoplasm where it is translated by ER‐bound ribosomes. TNF‐α polypeptide is then transported into the ER lumen by sec‐61 translocon. The protein is then folded and matured by ER chaperones, and processed under the quality control system (QCS) in the ER lumen. The correctly folded TNF‐α moves along the secretory pathway, and is transported to the Golgi apparatus where it folds into homotrimers before being delivered to the plasma membrane via the vesicle. TNF‐α on the cell membrane is retained in its membrane‐bound form or, after cleavage by TACE (TNF‐α converting enzyme), sheds a soluble form that acts on the TNF receptors (TNF‐R) of target cells. Misfolded or incompletely folded TNF‐α is retained in the ER or tagged for degradation by ER‐associated degradation (ERAD) or through ER‐phagy, thereby maintaining proteostasis and regulating functional TNF‐α for signalling. This figure was created using BioRender.com.

Consequently, pro‐TNF‐α monomers that fail to fold, form correct disulfides, or assemble into trimers are routed into ERAD so that only properly folded, trimeric TNF‐α exits the ER [43]. Co‐translationally, pro‐TNF‐α homo‐trimerises by specific protomer–protomer interfaces in the C‐terminal TNF homology domain, which adopts a jelly‐roll/β‐sandwich fold [3]. TNF‐α homotrimers traffics via COPII vesicles through the Golgi to the plasma membrane, where it functions in juxtacrine signalling or is shed by ADAM17 to generate soluble TNF‐α [44, 45] (Fig. 1).

### Endoplasmic reticulum stress & unfolded protein response (UPR)

Under inflammatory conditions, TNF‐α stimulation rapidly elevates intracellular reactive oxygen species (ROS), commonly via NADPH‐oxidase activation. ROS perturb the ER luminal redox environment required for oxidative folding, leading to the accumulation of unfolded or improperly folded nascent polypeptides that activate the UPR [46]. Accumulation of misfolded proteins induces principal ER‐membrane sensors, including IRE1α, whose auto‐phosphorylation promotes the splicing of XBP1 mRNA to generate the active transcription factor XBP1s. XBP1s then controls the transcription of genes encoding ER chaperones, PDIs, and ERAD components to expand folding and degradative capacity [19]. In primary human airway smooth muscle cells, exogenous TNF‐α increases IRE1α phosphorylation and XBP1 mRNA splicing in a dose‐ and time‐dependent manner, an effect dependent on ROS and mitigated by chemical or enzymatic ROS products [19]. Conversely, in THP‐1 monocytes, experimentally induced ER stress elevates canonical UPR markers (IRE1α, CHOP, and ATF6) and amplifies TNF‐α transcription and secretion through ROS/CHOP and MAPK–NF‐κB mechanisms [47]. Importantly, the accumulation of misfolded pro‐TNF‐α can activate IRE1α, which in turn upregulates GRP78/BiP, PDIs, and lectin chaperones to restore ER folding capacity and maintain secretory homeostasis [48]. This coordinated response transiently boosts ER folding capacity while linking proteostatic imbalance to increased inflammation. These findings indicate that activation of the UPR downstream of TNF‐α is attributable to inflammatory redox stress rather than a TNF‐specific endoplasmic reticulum signalling pathway. Comparable ROS‐dependent activation of endoplasmic reticulum (ER) stress pathways has been documented of other pro‐inflammatory cytokines, such as IL‐1β and IL‐6, suggesting that TNF‐α may enhance a more generalised proteostatic stress response. Nevertheless, it remains unclear whether TNF‐α selectively influences specific UPR branches or activates non‐canonical IRE1 signalling.

## TNF‐α trafficking and proteostasis control

### ER‐to‐Golgi transport: regulation by COPII vesicles

The secretion of important T cell–derived inflammatory cytokines, including TNF‐α, is central for the inflammatory signal transduction. Once correctly folded and assembled in the ER, pro‐TNF‐α trimers are packaged into COPII vesicles for anterograde transport to the ER‐Golgi intermediate compartment (ERGIC) and Golgi stacks. Subsequently, transmembrane protein TNF is delivered to specific sites on the cell surface via COPII‐mediated ER‐to‐Golgi transport [45]. Indeed, secretion is limited by SEC23 availability; and SEC23 deletion leads to intracellular retention of the secretome in T cells [45]. However, tubular or vesicular carriers move along microtubule tracks to their destinations, and they are responsible for the docking and fusion of the cargo at specific membranes by the Rab family of small GTPases and the SNARE family of membrane fusion proteins [49]. In LPS‐stimulated cells, TNF‐α protein accumulates primarily within the Golgi complex in activated cells and accumulates in intracellular compartments in response to IFN‐γ. This intracellular accumulation of TNF‐α may be a mechanism to ensure that release occurs only in concentrated pulses in response to a stimulus [50].

ER‐resident chaperones not only assist folding but also regulate cargo competence for export. One important ER chaperone, GRP78/BiP, is upregulated by UPR‐signalling cascades and is secreted out of the cell. At the extracellular space, GRP78/BiP mediates a range of anti‐inflammatory functions, which facilitate the timely resolution of inflammation while attenuating TNF‐α production [51]. Likewise, the Calnexin/calreticulin cycle ensures that only mono‐glucosylated, properly folded trimers progress to ER‐exit sites and promote TNF‐α secretion [52]; otherwise, misfolded proteins are retained and eventually targeted for ERAD. Overexpression of BiP or inhibition of the Calnexin cycle experimentally reduces TNF‐α surface trafficking, underscoring chaperone‐mediated quality control as a critical checkpoint upstream of COPII vesicle formation [51, 52].

#### Golgi processing & post‐translational modifications

Although TNF stimulation and secretion are regulated by intracellular signal cascades, post‐translational modifications (PTMs) possess an important regulatory mechanism, known to be involved in intracellular TNF‐protein trafficking. TNF is subjected to various PTMs such as phosphorylation, myristoylation, palmitoylation, and glycosylation (Table 1). At the ER/Golgi transition, pro‐TNF undergoes palmitoylation at a conserved cysteine near the transmembrane‐cytosolic boundary. While palmitoylation adds a lipid anchor that influences membrane microdomain localisation, it does not directly affect ectodomain shedding [53]. Recently, Murata et al. found that N‐glycosylation at residue Asn‐86 of TNF‐α downregulates its expression and secretion. Essentially, mutation of this site increases TNF‐α protein levels without affecting mRNA, indicating a post‐translational control mechanism [54]. While TNF‐α itself is not directly phosphorylated, its receptors—particularly TNFR1— are phosphorylated by various kinases, and these phosphorylation events are crucial for TNF signalling. For instance, phosphorylation of TRAF2 at Ser 11 regulates its capacity to induce anti‐apoptotic target genes downstream of full TNF‐α‐induced activation, which promotes NF‐κB pathway activation and renders cells resistant to stress‐induced apoptosis [55].

**Table 1:** Post‐Translational Modifications (PTM) of TNF‐α, site of modification, and functional roles.

PTM type	Residue	Functional role	References
Proteolytic cleavage	Ala^76^‐Val^77^ (pro‐TNF‐Α)	ADAM17 (Tumour Necrosis Factor α Converting Enzyme, TACE) cleaves the 26 kDa membrane protein to release the soluble ~17 kDa mature TNF‐α; essential for secretion and paracrine signalling	[55]
Palmitoylation	Transmembrane domain (Cys residues)	Lipid modification that influences localisation in lipid rafts affects interaction with TNFR1, signalling potency, and intracellular fragment turnover	[114]
Myristylation	Lys^19^, Lys ^20^	Affects membrane association and subcellular targeting	[115]
O‐GlcNAcylation	Ser^4^	Ser^4^ is located near the receptor‐binding interface of the TNF‐α trimer, so glycosylation could sterically influence receptor binding, especially in some isoforms.	[116]
Phosphorylation	Serines in the STES motif (N‐terminal cytoplasmic domain)	Mediates reverse signalling from TNF‐α when ligated by sTNFR. Involved in a rise in intracellular calcium following sTNFR binding.	[117]

### Conventional and non‐conventional protein secretion

#### Conventional secretion mechanism: shedding by ADAM17/TACE

The primary mechanism for converting membrane‐bound TNF‐α (pro‐TNF) into the soluble cytokine is through proteolytic cleavage by TNF‐α‐converting enzyme (TACE/ADAM17). These type I transmembrane metalloprotease catalyses bond cleavage at the Ala76‐Val77 junction within the TNF ectodomain. Experiments using mouse‐embryo fibroblasts missing ADAMs 9, 10, 17 or 19 showed that only the absence of ADAM17 prevented stimulated TNF‐α release [56]. New insights have advanced the understanding of ADAM17 regulation by emphasising the critical roles of post‐translational control and membrane signalling environments in determining substrate‐shedding efficiency [61, 62]. In macrophages, a newly synthesised TNF precursor traffics via COPII vesicles through the Golgi, transiently accumulating in the trans‐Golgi network, then moves in recycling endosomes to the plasma membrane by constitutive exocytosis [44]. TNF‐α trafficking and secretion are not random, but rather specifically delivered to sites of phagocytic cup formation. In activated macrophages, newly delivered surface TNF‐α was highly concentrated in the phagocytic cups, clustered along relevant secretion machinery at the delivery site, including the SNARE complex required for TNF‐α delivery, and TACE [63]. Not until the TNF precursor is at the cell membrane, TACE enzyme cleaves residues between alanine‐76 and valine‐77; therefore, the release of a 17 kDa soluble and mature TNF‐α homotrimers into the extracellular space [44]. This proteolytic processing is essential for TNF's paracrine and systemic activities. Essentially, TNF‐α maturation and trafficking depend on ADAM17 phosphorylation status, which controls its ability to process pro–TNF‐α into the mature, secreted cytokine. ADAM17's cytosolic tail phosphorylation, mediated by PKC and MAPKs, modulates its ability to cleave and further shed TNF‐α and TNF receptors [64].

#### Non‐conventional secretion mechanism: exosome‐mediated TNF‐α release

Exosome‐enclosed TNF‐α is an emerging pathway for cytokine transport and intercellular signal cascade. Upon inflammation, dendritic cells constitutively produce exosomes; essentially, they carry membrane proteins such as TNF‐α, a Fas ligand, and TNF‐related apoptosis‐inducing ligand, which effectively mediate caspase activation and apoptosis in tumour cells. Dendritic cell‐derived exosomes directly induce the activation of natural killer cells, mediated by the engagement of exosomes‐associated TNF‐α derived from dendritic cells, which interacts with natural killer cell TNFR1 and TNFR2 [65]. Furthermore, TNF‐α is found significantly increased in the serum extracellular vesicles of colorectal cancer cells, and tumour cell‐derived exosomes' TNF‐α strongly correlates with increased migration, invasion, and metastasis both in vitro and in vivo [66]. Interestingly, in activated immune cells, ATP redirects TNF trafficking pathways, switching off soluble TNF‐α (17 kDa) release from activated macrophages, while preferentially packaging transmembrane pro‐TNF‐α (26 kDa) within released microvesicles [67].

### TNF‐α degradation pathways

#### Proteasomal degradation

Inflammation has been widely associated with ER stress and subsequently upregulates ERAD transcription components, including ATF6α, PERK and IRE1α [68]. While TNF‐α expression is not only transcriptionally and post‐transcriptionally regulated, it is also dependent on proteostasis mechanisms that ensure its proper expression, folding and degradation. As such, PN would control the fate of both TNF‐α and its upstream regulators. TNF‐α has a relatively short half‐life, since it is immediately broken down and removed from circulation, as part of regulating TNF's effects and preventing prolonged inflammation. Activated immune cells—such as polymorphonuclear neutrophils—release elastase that can directly degrade TNF‐α in the extracellular environment, behaving as a local feedback mechanism to prevent excess TNF‐α‐induced inflammation [69].

Alternatively, the ubiquitin system is complex, multifaceted, and is tightly regulated at different levels by a range of enzymes, including E1s, E2s and E3s, and an array of deubiquitinating enzymes, which govern proteasomal degradation. During an inflammatory response, ubiquitination can either activate or dampen inflammasome activation, while controlling either protein stability, complex formation, or, in some cases, directly affecting receptor activity [70]. E3 ubiquitin ligases play a crucial role in the TNF‐α pathway by mediating the degradation of key signalling molecules, thus regulating the intensity and duration of the inflammatory response. Within the PN, E3 ligases mark target proteins with (poly)‐ubiquitin tags to promote their breakdown by the proteasome. Several E3 ligases, including those from the TRIM and cIAP families, have been identified as regulators of the TNF‐α‐induced NF‐κB pathway, either promoting or inhibiting its activity [71]. Importantly, E3 ubiquitin ligases serve as substrate‐specific gatekeepers in this system. Qian et al. have shown that the HECT‐domain E3 ligase Smurf1 downregulates USP5, a deubiquitinase critical for TNF‐α production. Through direct ubiquitination and proteasomal degradation, it integrates into the PN to modulate inflammatory cytokine output [72]. Disruption of this system can result in ER stress, prolonged TNF signalling, and unresolved inflammation, contributing to chronic inflammation [47].

#### Autophagy‐lysosome‐based degradation

Following activation, TNF receptors are endocytosed and are either recycled or degraded within lysosomes. This attenuation mechanism is closely linked to autophagic pathways. Upon ligand binding, TNFR1 undergoes a conformational change that unmasks internalisation signals and is able to recruit adaptor proteins, therefore promoting clathrin‐dependent endocytosis [73]. Subsequently, receptors destined for signal termination are sorted into intraluminal vesicles of multivesicular bodies and fuse with lysosomes, where TNFR1 is degraded, thereby attenuating further signalling. Essentially, since internalisation is central for TNF receptosomes to mediate apoptotic pathways (e.g. TRADD), its blocking in U937 cells prolongs NF‐κB activation and protects against TNF‐induced apoptosis [74]. For example, when TNFR1 is mutated, autophagy becomes impaired, leading to increased inflammation in conditions such as TNF‐receptor‐associated periodic syndrome [75]. Notably, in stress conditions, the regulation of TNF‐α‐induced autophagy is disrupted. Under conditions of chronic neuroinflammatory stress, TNF‐α impairs lysosomal acidification and autophagic flux in neuronal cells, leading to p62 accumulation, a protein involved in autophagosome formation and maturation, RIPK1 oligomerisation, and enhanced downstream necroptotic signalling [76].

## TNF‐α signalling and proteostasis crosstalk

### TNF‐α‐induced NF‐κB activation

**Fig. 2:**
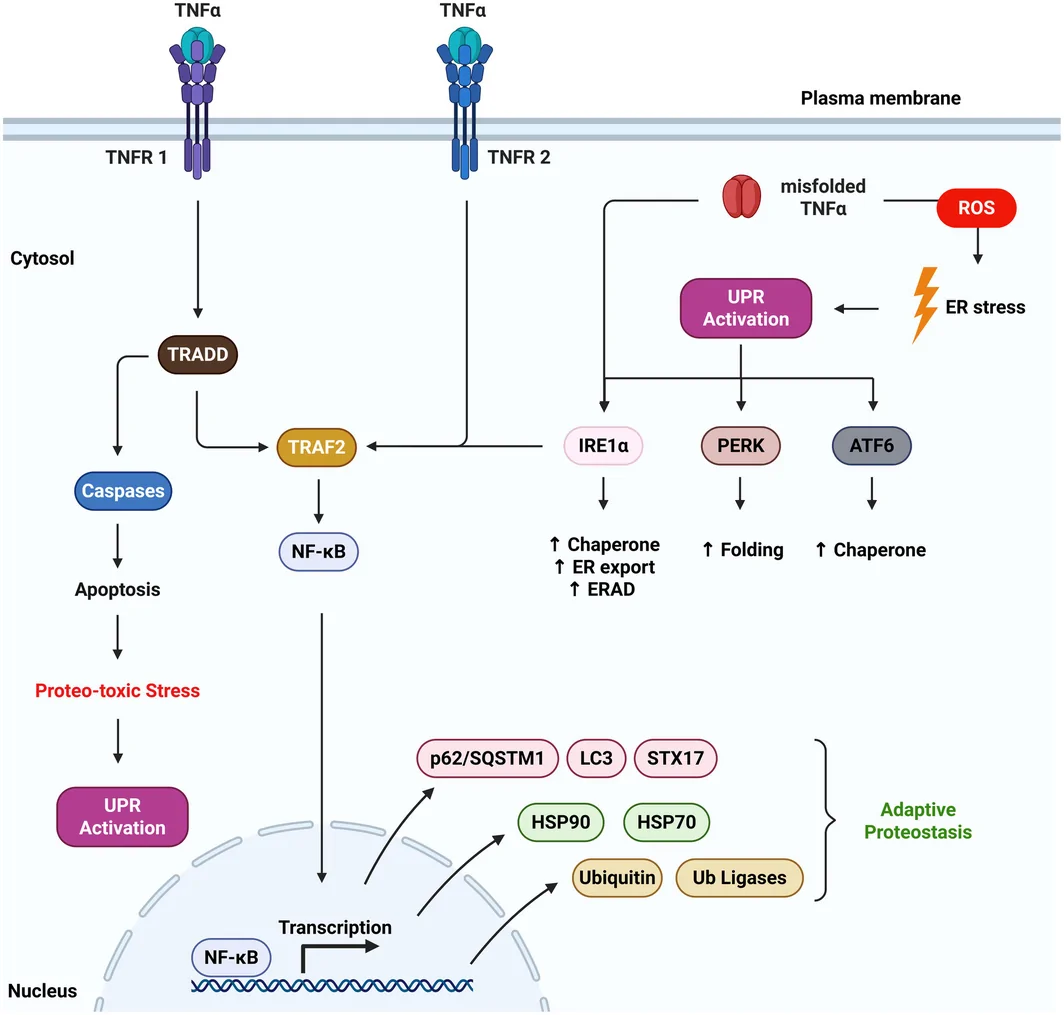
TNF‐α signalling, UPR and proteostasis crosstalk. TNF‐α binds to the TNFR1 trimer on the cell surface and recruits the adaptor proteins TRADD and downstream caspases, triggering apoptosis and proteotoxic stress, which can secondarily activate the unfolded protein response (UPR). In contrast, TNF‐α engagement with both TNFR1 and TNFR2 recruits TRAF2, which in turn triggers the canonical NF‐κB pathway. Once in the nucleus, NF‐κB upregulates stress response genes implicated in protein folding, including molecular chaperones such as HSP70 and HSP90, components of the ubiquitin–proteasome system, and autophagy receptors such as p62/SQSTM1 and LC3, and thereby supporting adaptive proteostasis. However, accumulation of misfolded TNF‐α or ROS can trigger ER stress and thus activation of UPR sensors IRE1α, PERK and ATF6, which enhances folding capacity, expression of chaperones, ER‐associated degradation (ERAD), and protein export. Together, these processes integrate TNF‐α signalling with ER stress responses to maintain a balance between cell survival, inflammation, and proteostasis. This figure was created using BioRender.com.

TNF‐α binding to TNFR1 triggers the canonical NF‐κB pathway. When TNF‐α binds to the TNFR1 trimer on the cell surface, it recruits the adaptor proteins TRADD, kinase RIP1, and TRAF2, along with the E3 ligases cIAP1/2, to form membrane‐associated Complex I. TRAF2 and cIAP1/2 catalyse K63‐linked and linear ubiquitination of RIP1, generating docking sites for the TAK1 kinase complex and the IKK complex (composed of IKKα, IKKβ and NEMO/IKKγ), with LUBAC contributing linear ubiquitin chains to stabilise the signalling cascade [77]. Activated IKKβ phosphorylates the inhibitor IκBα on Ser^32^/^36^, marking it for SCF^βTrCP^‐mediated polyubiquitination and rapid proteasomal degradation (Fig. 2). This releases the NF‐κB p65/p50 heterodimer, which translocates to the nucleus and drives transcription of inflammatory and survival genes, such as cytokines, chemokines, and anti‐apoptotic factors [78].

#### Exploitation of the ubiquitin‐proteasome system

The ubiquitin‐proteasome system is essential not only for signal propagation but also for basal protein quality control. Under inflammatory stress, increased proteasome activity clears both regulatory proteins (IκBα) and misfolded proteins. The proteasome‐mediated clearance of IκB releases NF‐κB (p50/p65) dimers to translocate into the nucleus. Conversely, disrupting the function of the proteasome, for example, by inhibitors like bortezomib or MG‐132, stabilises IκBα and blocks NF‐κB activation [79].

#### Transcriptional reinforcement of proteostasis networks

While a 5‐min TNF stimulation can activate the NF‐kB signalling pathway, imposing its transcriptional effect requires a more extended or repeated stimulus [80]. After a sustained stimulus, and once in the nucleus, NF‐κB upregulates a suite of genes implicated in protein folding, including molecular chaperones such as HSP70 and HSP90 [81], components of the ubiquitin–proteasome system (e.g. ubiquitin itself, E2 conjugating enzymes, and E3 ligases like MuRF1 in muscle) [82], and autophagy receptors such as p62/SQSTM1 and LC3 [83]. Notably, NF‐κB directly contributes to the expression of heat‐shock proteins, primarily HSP70 (HSPA1A), by binding to a κB element in its promoter. The resulting increase in HSP70 levels enhances folding capacity and assists in the degradative pathways of misfolded proteins, thereby preventing aggregation [84]. Under TNF‐α‐stimulated conditions, NF‐κB activation not only drives inflammatory gene expression but also reinforces the PN and coordinates signalling mechanisms. While TNF‐α‐induced activation of the canonical NF‐κB pathway leads to increased transcription of genes encoding the proteasome regulatory subunit S5b/PSMD5, TNF‐α/NFκB inhibits 26S proteasome assembly and thus reduces proteasome activity [85]. In addition, prolonged TNF‐α exposure activates macro‐autophagy, and NF‐κB itself transcriptionally controls key proteostasis effectors (IκBα, A20, BAG3, proteasome subunits) [86]. Through these transcriptional programmes, TNF‐α‐induced NF‐κB activation dynamically adjusts proteasomal activity, autophagic turnover, and molecular chaperone expression to regulate protein homeostasis during inflammatory stress. However, its dysregulation has been linked to chronic inflammatory and neurodegenerative disorders.

#### Termination of TNF‐α‐induced NF‐κB signal transduction

TNF‐α‐induced NF‐κB activation is a transient mechanism, activated upon some external stimulus. Once the stimulus is withdrawn, TNF‐α‐bound TNFR1 is internalised via clathrin‐mediated endocytosis and directed to lysosomes for degradation. This removes the initiating signal and prevents persistent proteasomal degradation of IKK, and subsequent NFkB activation [87]. Intriguingly, NF‐κB promotes transcription of IκBα, a negative feedback mechanism that terminates this signalling pathway [88]. Additionally, A20 is rapidly induced by NF‐κB and terminates signalling by removing K63‐linked ubiquitin chains from RIP1, then promoting RIP1 K48‐ubiquitination (and degradation) through its zinc‐finger E3 ligase activity [89]. As a result, such mechanisms terminate NF‐κB signalling and allow periodicity of TNF‐α‐induced NF‐κB activation and limit excessive expression of inflammatory and stress‐response genes. NF‐κB activation induced by TNF‐α exerts proteostatic effects primarily through transcriptional reprogramming of stress response and degradation pathways. Consequently, most observed proteostatic changes under TNF‐α stimulation are likely secondary effects of the inflammatory response, rather than direct regulation of the protein folding machinery. However, whether TNF‐α alone controls proteostasis via NF‐κB signalling or if other inflammatory cytokines employ similar regulatory mechanisms, remains poorly understood.

### TNF‐α‐mediated modulation of protein aggregation and stress responses

TNF‐α plays a multifaceted role in tuning cellular stress responses by influencing the formation and clearance of protein aggregates, chiefly through stress granule (SG) dynamics, ROS generation, and ER stress pathways. Recent experimental studies demonstrate that stress granules dynamically reorganise mRNAs and RNA‐binding proteins to coordinate translational arrest and facilitate cellular adaptation during inflammatory stress [90, 91]. A TNF‐α pro‐inflammatory signal can rapidly trigger the phosphorylation of eIF2α, via PKR or PERK kinases, leading to global translational arrest and the nucleation of SGs, upon which untranslated mRNAs and misfolded proteins are sequestered into dynamic, membrane‐less assemblies [92].

In colonic epithelial cells, Hu et al. showed that under heat‐shock conditions, TNF‐α might increase SG formation and simultaneously suppress Hsp70 translation, as part of TNF‐α modulation for proteostasis [93]. Moreover, TNF‐α activation of NF‐κB and MAPK pathways also promotes mitochondrial and NADPH‐oxidase‐derived ROS, reinforcing proteotoxic stress [94]. Stimulating L929 fibrosarcoma cells with TNF‐α triggers PERK‐mediated phosphorylation of eIF2α, ATF6α cleavage to p50ATF6, and IRE1α‐dependent splicing of XBP1 mRNA. This induction was strictly dependent on ROS accumulation. In turn, the UPR system mitigates TNF‐α‐induced ROS accumulation and cell damage by enhancing glutathione biosynthesis and folding capacity [46]. In addition, TNF‐α disrupts autophagic clearance mechanisms. In platelets and megakaryocytes, elevated TNF‐α levels impair autophagic flux by downregulating syntaxin 17 (STX17), a SNARE protein essential for autophagosome–lysosome fusion. Loss of STX17 impairs autophagic flux and leads to the accumulation of dysfunctional mitochondria and aggregated proteins. Pharmacologic blockade of TNF‐α restores STX17 expression, rescues autophagy/mitophagy, and normalises cellular metabolism [95]. Together, these mechanisms illustrate that TNF‐α not only promotes inflammatory signalling, but its abnormally elevated levels might participate in disrupting PN and the accumulation of potentially toxic protein aggregates in diseases. Despite this, it is still unclear if TNF‐α directly regulates stress‐granule dynamics or if these effects are primarily due to general inflammatory stress responses observed with other cytokines.

## Dysregulation of TNF‐α‐proteostasis in disease

TNF‐α is a pleiotropic cytokine whose levels and activity are tightly regulated at the levels of synthesis, folding, PTM, and degradation, and has emerged as a key modulator of cellular PN. Under physiological conditions, this network ensures that TNF‐α is produced and cleared in a manner that finely balances host defense with tissue homeostasis. However, upon sustained or dysregulated TNF‐α exposure, TNF‐α may contribute directly to the pathogenesis of chronic inflammatory diseases, cancer and neurodegeneration.

### Neurodegeneration: Alzheimer's, ALS, and Parkinson's

In response to defective proteostasis, misfolded proteins are deposited and detected in biofluids, so that they can be identified as potential biomarkers for early diagnosis of misfolded‐protein diseases. Sustained TNF‐α elevation in neurodegenerative disorders undermines neuronal proteostasis by exacerbating ER stress and maladaptive UPR signalling. In Alzheimer's disease, TNF‐α amplifies ER stress‐induced activation of PERK‐eIF2α and CHOP, promoting both amyloid‐β accumulation and tau hyperphosphorylation. These aggregates then interfere with normal processes, exacerbate ER stress, and participate in the neuroinflammation pathology [96]. In Parkinson's disease, emerging evidence links elevated TNF‐α to increased levels of ER stress markers (GRP78, CHOP, XBP1s) in dopaminergic neurons, where proteostasis imbalance accelerates α‐synuclein aggregation and cell death [97]. Although less well characterised, in amyotrophic lateral sclerosis (ALS), autocrine TNF‐α signalling has been shown to trigger IRE1α‐mediated UPR activation and sensitise motor neurons to ER stress, as well as enhancing astro‐ and microgliosis in the promotion of neuroinflammation. Ablation of IRE1α downstream effector XBP1s in the ALS‐mouse model upregulates the autophagy pathway, promotes aggregate degradation, and slows disease progression [21].

### Inflammatory diseases

In rheumatoid arthritis (RA), characterised by inflammation of the synovial membrane lining of the joints, fibroblasts greatly expand in number via the TNFα‐secreted by macrophages. Synovial fibroblasts are relatively resistant to ER stress‐induced apoptosis in attribution to hyper‐ERAD and the expression of synoviolin 1 (SYNV1, HRD1), the TNFα‐inducible E3 ubiquitin ligase. While fibroblasts use both the proteasome and lysosome/autophagy pathways to clear excess protein and promote survival, TNF‐α induces a partial ER stress response and sensitises them to proteasome inhibition, while simultaneously upregulating autophagy as a compensatory clearance pathway [98]. Inflammatory bowel disease (IBD) is characterised by chronic intestinal inflammation. Studies have consistently shown that TNF‐α plays a pathogenic role in the disease, as its elevated levels in the mucosa and serum can promote intestinal infiltration. Abnormal TNF‐α levels in intestinal epithelial cells cause ER stress and mucin secretion, which lead to the disassembly of tight junction and adherence junction proteins, further worsening infiltration [99]. Remarkably, excessive TNF‐α mediates epithelial cell death in the absence of autophagy, in a mechanism involving mitochondrial ROS accumulation [100]. Similarly, in psoriatic keratinocytes, elevated levels of TNF‐α are tightly correlated with ER stress and the activation of the UPR system components. TNF‐α‐induced increased expression of ERS‐associated proteins: GRP78/BiP, CHOP and XBP1s in the Psoriasis vulgaris epidermis results in the progression of psoriasis pathogenesis and heightened inflammation [101].

### Cancer

TNF‐α is primarily produced by activated macrophages and T cells and, in cancer, it exhibits a dual role. At high acute concentrations, TNF‐α can induce apoptosis and necrosis of tumour cells via death‐receptor signalling, enhance infiltration of cytotoxic immune cells, and disrupt tumour vasculature. On the contrary, chronically elevated, low‐grade TNF‐α promotes tumour initiation and progression by activating NF‐κB and AP‐1 pathways, which mediate expression of cell proliferation, survival, angiogenesis, and metastatic molecules, as well as create an immunosuppressive microenvironment [102].

Many tumours utilise TNF‐α‐induced IκBα ubiquitination and proteasomal degradation, and subsequent activation of NF‐κB, where pathological levels of TNF‐α in the tumour microenvironment sustain constitutive NF‐κB activity observed in breast, colorectal, prostate, pancreatic, and many other cancers [81]. Consequently, this mechanism leads to the upregulation of anti‐apoptotic genes (e.g. BCL‐XL, cIAPs), promotes proliferation and angiogenesis, and drives chemoresistance [103]. Since activation of NF‐κB requires the ubiquitin‐proteasome system to degrade IκBα, proteasome inhibitors (e.g. bortezomib) block IκBα degradation and thus, NF‐κB activation [104].

Importantly, in the tumour microenvironment, TNF‐α engagement of TNFR1 activates TRAF2, which directly interacts with the IRE1α to activate the JNK pathway [105]. Tumour cells under ER stress (due to hypoxia, aneuploidy, high secretion load) thus face additional proteotoxic pressure when exposed to TNF‐α, forcing the switch from adaptive UPR to cell death pathways. Sustained TNF‐α exposure activates the canonical UPR sensors in tumour cells promoting upregulation of ER chaperones (e.g. GRP78/BiP) and protein‐folding machinery to cope with increased secretory demands [106]. For example, TNF secreted by activated inflammatory macrophages stimulates compensatory hepatocyte proliferation and expands hepatocellular cancer progenitors. TNF further reinforced ER stress, as evidenced by the elevation of ER stress‐related components, including CHOP, GRP78/BiP, XBP1s, p‐eIF2α, and p‐IRE1α [107]. Macrophage proteomics show that TNF‐α downregulates mitochondrial, proteasomal subunits, and chaperone proteins (e.g. HSP90), and alters the ubiquitin‐proteasome machinery, suggesting modulation of proteostasis, mitophagy, and protein degradation pathways [108]. In multiple myeloma, bone marrow stromal cells secrete TNF‐α in the tumour microenvironment to provoke NF‐κB cascades and induce autophagy via LC3, which also binds fibronectin mRNA, increasing its expression [109]. Moreover, TNF‐α reshapes the ubiquitin‐proteasome system in tumour cells by inducing the immunoproteasome, a specialised form of the 20S proteasome characterised by the catalytic subunits PSMB8, PSMB9, and PSMB10. The expression of these subunits is TNF‐α‐promoted through NF‐κB and AP‐1 pathways. Importantly, not only do immunoproteasomes participate in protein clearance, but can also, when deregulated, mediate cancer onset, progression, and therapy resistance [110]. This adaptive UPR permits malignant cells to withstand hypoxia, nutrient deprivation, and oxidative stress, while sending pro‐survival signals that allow tumour growth, metastasis, and resistance to therapies.

## Therapeutic targeting of TNF‐α and proteostasis

Biologic TNF‐α inhibitors, such as TNF‐α monoclonal antibodies (e.g. infliximab and adalimumab), suppress TNF‐α by blocking its cell surface receptors; therefore, they reduce TNF‐α‐induced chronic inflammation in diseases like RA and IBD, and help to restore the proteostatic balance [111]. Similarly, RA patients receiving anti‐TNF exhibit decreased synovial expression of autophagy‐related (LC3‐II) and citrullinated proteins [112]. Therefore, this suggests that TNF‐α neutralisation can restore balanced protein turnover and reduce the effects associated with accumulated proteins [110]. Furthermore, TNF‐α‐based therapeutics have demonstrated efficacy in treating various inflammatory disorders, establishing TNF‐α blockade as a well‐validated therapeutic strategy, helping millions of patients to cope with disease. However, some patients may eventually become resistant to those treatments. As an addition to TNF‐α‐based treatments, the combination with proteostasis modulators is still at the preclinical stage. For instance, in preclinical collagen‐induced arthritis (CIA) in rats, delanzomib, a proteasome inhibitor, combined with adalimumab, an anti‐TNF‐α drug, produced superior anti‐arthritic efficacy and more strongly blocked TNF‐α‐induced inflammation than either agent alone [113]. Chemical chaperones, as 4‐phenylbutyrate (4‐PBA), were associated with reduced expression levels of ER stress markers, PERK (phosphorylation), ATF6 (proteolytic cleavage), XBP1 (mRNA splicing) and lower mucosal expression of chaperones (GRP78/BiP) by attenuating UPR activation and lowering TNF‐α release from epithelial cells in Crohn's disease [114]. Furthermore, anti‐chaperone therapy has emerged as a novel therapeutic approach, and is aimed at relieving aberrant inflammatory responses. For instance, HSP90 inhibitor 17‐AAG has been reported as reducing TNF‐α (alongside IL‐1β and MMP‐13) in vivo, by simultaneously ameliorating the inflammatory phenotype of macrophages [115]. Agents such as rapamycin and MG‐132 modulate PN since MG‐132 is an inhibitor of the ubiquitin‐proteasome system, and rapamycin induces autophagy. Combining both treatments not only significantly suppressed cytoplasmic expression of TNF‐α, but also transformed the intensive immune response into immune tolerance [116]. Preclinical cancer studies demonstrate that enhancing autophagy has been shown to sensitise tumour cells to TNF‐α‐targeting therapies by reducing the proteotoxic burden.

Beyond global TNF neutralisation, selective modulation of TNF‐α/TNFR signalling rebalances proteostasis while preserving normal immune functions. Novel strategies include engineered TNF‐blocking peptides that preferentially inhibit TNFR1 (pro‐inflammatory) while sparing TNFR2 (regeneration). In animal models, deletion of TNFR1 is associated with reduced disease phenotype, while TNFR2 deletion has the opposite effect [117]. Intervening at the very earliest steps of protein biogenesis could be a powerful complementary strategy to TNF‐α neutralisation. By enhancing co‐translational quality control and ER translocation, one could reduce the generation of aberrant secretory/membrane proteins that trigger the UPR and downstream TNF‐driven inflammation. Modulating the integrated stress response can recalibrate translation to a level that relieves proteostatic pressure without globally suppressing host defenses. Bhattacharya et al. showed that prolonged TNF‐αstimulation could trigger the JNK/IFN‐β/PKR circuit that drives eIF2α phosphorylation and an integrated stress response (ISR) following infection‐induced inflammation. Importantly, ISR inhibition reduces associated necrosis, supporting upstream modulation of proteostasis as a host‐directed strategy. In the same model, blocking translation with ‘Rocaglate’ derivatives or preventing ROS‐driven protein aggregation upon priming macrophages with antioxidants (e.g. BHA), boosts antioxidant defense before TNF stimulation, prevents protein aggregation, and inhibits super‐induction of both heat shock proteins and proteostatic flux upstream of the UPR/ISR [118]. Although the combination therapy offers a promising therapeutic strategy, it involves a systemic targeting of core proteostasis pathways that plays a critical role in maintaining cellular homeostasis. The proteasome, autophagic flux, and translational regulation are essential processes operating across tissues, and broad pharmacological modulation of these pathways may lead to off‐target toxicity, impaired immune competence and unintended tissue damage. In addition, given that most combination strategies are primarily supported by preclinical data, it is crucial to take a careful assessment of their safety, dosing, and tissue specificity before they can be applied in clinical trials. Because TNF‐α biogenesis is controlled at multiple steps, early interventions targeting TNF‐α biogenesis provide an upstream level to limit its production and downstream inflammation. Translationally, eIF5A could serve as a target for anti‐inflammatory drugs, since it regulates ribosomal function that controls the translation of TNF‐α mRNA and subsequent proinflammatory response [119]. In addition, partial and selective control of ER translocation (Sec61/SRP axis) offers another conceptual targeting to TNF‐α and TNF‐α‐associated inflammation, since actively translating polypeptides are associated with the ER. Mycolactone, a toxin produced by the bacterium *Mycobacterium ulcerans*, can block TNF‐α translocation and thereby suppress its secretion [57]. These experiments serve as a proof‐of‐principle for targeting pre‐ or post‐translational machineries to block TNF‐α expression and TNF‐α‐induced chronic inflammation. Practically, these upstream strategies could be combined with TNF pathway‐selective agents (e.g. TNFR1‐biased blockers) to: [1] reduce the feed‐forward generation of proteotoxic signals, [2] lower tissue TNF production, and [3] permit lower doses of systemic TNF blockade, potentially preserving regenerative TNFR2 biology.

## Conclusion and future perspectives

The intricate and bidirectional interplay between TNF‐α signalling and the cellular PN relates health and disease and explains how disruption of this balance results in inflammation, cancer, or neurodegeneration. Emerging evidence shows that TNF‐α not only depends on proteostasis components (e.g. chaperone‐mediated folding, ERAD, UPR, and autophagy) for its biogenesis, maturation, and clearance, but can also impact proteostasis pathways through its downstream inflammatory and stress‐responsive signalling cascades. Under homeostatic conditions, TNF‐α ensures a balanced inflammatory response and maintains protein quality control systems. However, chronic or deregulated TNF‐α level drive sustained and pathologic ER stress, UPR activation, and autophagy, leading to aggregation of misfolded proteins, which contribute to disease development.

Despite these insights, the crosstalk between TNF‐α and PN requires further investigation. Indeed, the specific mechanistic pathways needed for pro‐TNF‐α folding and maturation within the ER remain incompletely defined. Additionally, the dual role of TNF‐α in promoting or suppressing proteostasis components (quality control enhancing, ER stress, UPR overactivation, and autophagy overload), upon acute or chronic stimulation, is yet to be defined. From a translational point of view, managing proteostasis via targeted therapies for TNF‐α‐driven diseases holds promise. Further, advances in single‐cell proteomics, ubiquitin regulation, and conditional CRISPR‐based systems will deepen our understanding of the TNFα‐proteostasis crosstalk.

## Conflict of interest

EC is founder of Thabor Tx and of Exa Noma Therapeutics. The other authors declare that they have no conflict of interest.

## Author contributions

B.H. performed the literature search and wrote the manuscript. C.P., E.C., and J.Z. contributed to manuscript editing, interpretation of the literature, and critical revision. J.Z. conceived the review and supervised the manuscript preparation. All authors read and approved the final version of the manuscript.
